# Matrix metalloproteinases are important for follicular development in normal and miniature pigs

**DOI:** 10.1007/s10529-014-1474-9

**Published:** 2014-02-22

**Authors:** Sang Hwan Kim, Cher Won Kang, Kwan Sik Min, Jong Taek Yoon

**Affiliations:** 1 Institute of Genetic Engineering, Hankyong National University, Ansung, Gyeonggi-do 456-749 Korea; 2 Graduate School of Bio and Information Technology, Hankyong National University, Ansung, Gyeonggi-do 456-749 Korea; 3 Department of Animal Life Science, Hankyong National University, Ansung, Gyeonggi-do 456-749 Korea

**Keywords:** Follicular development, In-situ zymography, Matrix metalloproteinases (MMP), Miniature pig, Tissue inhibitors of MMPs

## Abstract

To determine the differences in the follicular development process of normal and miniature pigs, we compared the expression of matrix metalloproteinases (MMP) in these two breeds throughout folliculogenesis. In normal pigs, MMP-9 was highly expressed in all stages of the follicular development as well as during ovulation. The follicular development exhibited strong gelatinase activity. The expression of MMP-2 remained insignificant throughout folliculogenesis in both breeds. However, for the follicles of miniature pigs, MMP-2 level was higher than that in normal pigs. Thus MMP types may regulate the remodelling of follicular tissue in the ovaries of normal and miniature pigs. The differential expression of MMPs observed in this study reflected the mechanisms underlying ovarian follicular development in these two breeds.

**Electronic supplementary material:**

The online version of this article (doi:10.1007/s10529-014-1474-9) contains supplementary material, which is available to authorized users.

## Introduction

Miniature pigs are a breed that was developed and is widely used for medical research, including research in toxicology, pharmacology, and aging. Possibly because these animals are highly inbred, their reproductive characteristics, including ovulation rate and litter size, are lower than those of normal pigs (Park et al. 2006). The number of follicles and *corpora lutea* in miniature pigs is smaller than in normal pigs and reduced angiogenesis and delayed oocyte maturation may contribute to the smaller litter size of miniature pigs (Anderson 1997). Follicle development, which is important for proper maturation of oocytes and subsequent ovulation, can be divided into four phases: primordial, early, intermediate, and late. Upon stimulation with follicle-stimulating hormone (FSH), changes in the profile of hormones secreted by the granulosa cells and theca cells induce follicle expansion and oocyte maturation. Hormonal imbalance and other problems, such as follicular atresia and follicular cysts, may contribute to the small litter size of miniature pigs (Hirshfield 1991). Attempts have been made to overcome these problems by genetically modulating the expression of apoptosis-associated genes in the developing ovaries of miniature pigs (Kim et al. 2012). Follicle development in the ovary follows a major reorganization of ovarian tissue. The pivotal part of this reorganization is the changes in the extracellular matrix (ECM) components (Greenwald and Roy 1994). In this process, type VI collagen, the main ingredient of the basement membrane, is degraded by matrix metalloproteinases (MMPs): MMP-2 and MMP-9 (Visse and Nagase 2003). MMP-9 is a 92 kDa collagenase/gelatinase residing in the basement membrane of the ECM; it plays an important role in the penetration of trophoblasts (Ashida et al. 1996). MMP-2, another gelatinase in the ECM, cleaves collagens type V and VI, elastin, fibronectin, laminin, and gelatin. MMP-2 also facilitates the activation of MMP-9, thus regulating the development of follicles. The activity of MMPs is, in turn, controlled by a family of specific endogenous tissue inhibitors of metalloproteinases (TIMPs), which inhibit MMP enzymatic activity (Visse and Nagase 2003). Together, MMPs and TIMPs orchestrate the process of the ECM remodelling and regulate follicular growth and ovulation, as well as the development and regression of the *corpora lutea* (Goldberg et al. 1996).

The spatial expression of gelatinases in bovine and swine follicles probably reflects the distinct role of each enzyme in the physiological events of ovarian development (Basini et al. 2011). In this study, we have determined the expression pattern of the two key gelatinases MMP-9, which is involved in cell growth, and MMP-2, which is associated with cellular reorganization during follicle development and have compared the differences in MMP and TIMP expression between normal and miniature pigs. Our results show that during folliculogenesis, distinct types of MMPs and TIMPs are expressed in the ovaries of normal and miniature pigs. We believe that the differential expression of MMPs and TIMPs in these breeds may provide an insight into the molecular basis of reduced fertility in miniature pigs.

## Materials and methods

### Collection and preparation of ovarian tissue

Ovarian tissue samples were harvested following the method of Kim et al. (2012). Ovaries of five normal Landrace pigs (age: 10 months, weight: 110 ± 5.1 kg; Farm Animal, Ansung, Korea) and five miniature pigs (age: 10 months, weight: 26 ± 2.1 kg; Medi Kinetics Co., Ltd. Pyeongtaek, Korea) were collected on day 15 of the oestrous cycle from a local slaughterhouse at Pyeong-Nong, Pyeongtaek, Korea. The samples were placed into a freezer box in liquid N_2_ and transported to the laboratory within 2 h. Follicles at different developmental stages were separated and designated as developing, Graafian, and ovulated follicles according to the standards described by Stocco et al. (2007). Experiments were conducted in strict compliance with the recommendations specified in the Guide for the Care and Use of Laboratory Animals issued by the National Institutes of Health. The protocol was approved by the Committee on the Ethics of Animal Experiments of Hankyong National University (Permit Number: 2013-1).

### RNA extraction and real-time PCR

Deep-frozen ovarian tissues were treated with RBC lysis buffer (0.15 M NH_4_Cl, 1 mM KHCO_3_, and 0.1 mM EDTA) to remove red blood cells, homogenised, and centrifuged for 10 min at 3,000×*g*. Total RNA was extracted from the follicles using TRIzol, treated with DNAse according to the supplier’s instructions, and quantified by UV spectrophotometry. RNA purity was determined from A_260_/A_280_; a value between 1.8 and 2 indicated an RNA purity suitable for further analysis. First-strand cDNA was synthesised by reverse transcription of mRNA using the Oligo (dT) primer and Super Script II Reverse Transcriptase (Invitrogen). Real time (RT)-PCR analysis of 25 μl reaction mixture containing SYBR Green was conducted using the LineGene K system (Bioer Technology, Tokyo, JPN). The primers used for RT-PCR are listed in Supplementary Table 1. The Rotor-Gene Real-Time Software 6.0 was used to analyse the results, and the cycle threshold (Ct) values plotted in logarithmic scale were used to compare RNA expression. Gene expression levels relative to GAPDH (a house-keeping gene) were calculated using the 2-ΔΔCt method.

### Western blot analysis

Ovarian tissue protein extracts (30 μg) were separated by SDS-PAGE using 13 % (v/v) gels and transferred onto a PVDF membrane. Membranes were blocked with 5 % (v/v) non-fat dry milk overnight at 4 °C and then washed for 10 min with the washing buffer (0.1 % v/v Tween 20, 50 mM Tris/HCl, and 200 mM NaCl; pH 7.6). The membranes were incubated for 2 h with primary antibodies (dilution 1:1,000 in blocking buffer) that specifically recognised the active form of TIMP-2 and (Santa Cruz Biotechnology Inc., Texas, USA), TIMP-3, (Santa Cruz), and β-actin (Santa Cruz). After primary antibody binding, the membranes were washed three times for 15 min each with TBS-T buffer and incubated for 2 h with HRP-conjugated anti-goat (Abcam, Cambridge, UK) or anti-mouse (Abcam) secondary antibodies diluted 1:5,000 in blocking buffer). The membranes were incubated for 5 min with the ECL detection reagent in the dark and then exposed to X-ray film for 10 min; protein expression was normalized to that of β-actin, which served as an internal control, using the software Alpha Innotech ver. 4.0.0 (San Leandro, CA, USA).

### Zymography

To detect the gelatinase activity of MMPs, 20 mg total ovary proteins were mixed with 2 μl FOZ loading buffer (5 % Bromophenol Blue, 10 % v/v SDS, 2 % v/v glycerol in distilled water) on ice for 5 min. The sample was then subjected to SDS-PAGE in the gels containing 100 mg gelatin/ml for 90 min at 150 V. After electrophoresis, the proteins were renatured twice for 20 min in renaturation buffer (2.5 % Triton X-100 in PBS), and the gel was washed with sterile water for 20 min. After renaturation, samples were incubated in zymography reaction buffer (1 M Tris/HCl, 5 M NaCl, 1 M CaCl_2_, 0.2 mM ZnCl_2_, 0.2 % Triton X-100, and 0.02 % NaN_3_ in PBS; pH 7.5) at 37 °C for 18 h. Finally, the zymography gel was stained with Coomassie Brilliant Blue for 1 h to reveal clear areas of digested gelatin. The levels of active MMPs were assessed by western blotting.

### In situ zymography

Areas with gelatinase activity were detected by in situ zymography as previously described (Khandoker et al. 2001; Nemori and Tachikawa 1999). GN film (Fuji Photo Film Co.) coated with gelatin base emulsion was used to detect the site of gelatinase activity in the underlying tissue. Unfixed 7-μm-thick ovarian cryosections were mounted onto the GN film, incubated for 24 h at 37 °C, and stained with Bearish Scarlet (Chroma-Gesellschaft, mbH & Co., Munster, Germany) and hematoxylin. Specific pattern of gelatin digestion was indicated by white colour due to weaker staining with BS.

### Immunohistochemistry

Immunohistochemical detection of MMPs and TIMPs was carried out on 5 μm tissue sections mounted on siliconized slides. Briefly, paraffin sections were dewaxed with xylene substitute (Polysciences, PA, USA) and rehydrated in a graded series of ethanol. Antigen retrieval was performed by heating at 95 °C in 10 mM sodium citrate (pH 6.0). Endogenous peroxidases were quenched with 0.3 % H_2_O_2_ in methanol for 5 min at room temperature. After 3 washes in PBS, the slides were incubated in a blocking buffer containing 1 % (v/v) goat serum and 3 % (v/v) horse serum in PBS for 1 h at room temperature. Sections were incubated overnight at 4 °C with antibodies (diluted 1:150 in blocking buffer) against MMP-2 (Abcam), MMP-9 (Santa Cruz), TIMP-2, and TIMP-3. Sections were washed in PBS and incubated with secondary anti-rabbit, (Santa Cruz), anti-mouse (Santa Cruz), anti-goat, or anti-FITC HRP-conjugated (Takara, Osaka, Japan) antibodies (diluted 1:300 in blocking buffer) for 1 h at room temperature, rinsed, and developed for 10 min using the ABC detection kit (Vector, CA, USA); diaminobenzidine (Vector) was used as a substrate for HRP. Sections were counterstained with periodic acid–Schiff reagent and Harris hematoxylin solution containing 4 % (v/v) acetic acid. Tissues were dehydrated, cleared, and covered with Permount solution (Fisher, NJ, USA).

### Statistical analysis

Data were analysed with *t* test and general linear model (GLM) using the Statistical Analysis System software (SAS Institute, version 9.4, Cary, NC, USA). All experiments were repeated at least three times, and statistical significance was established at *P* < 0.05.

## Results

### Expression pattern of MMP and TIMP mRNAs in the ovaries of normal and miniature pigs

**Fig. 1:**
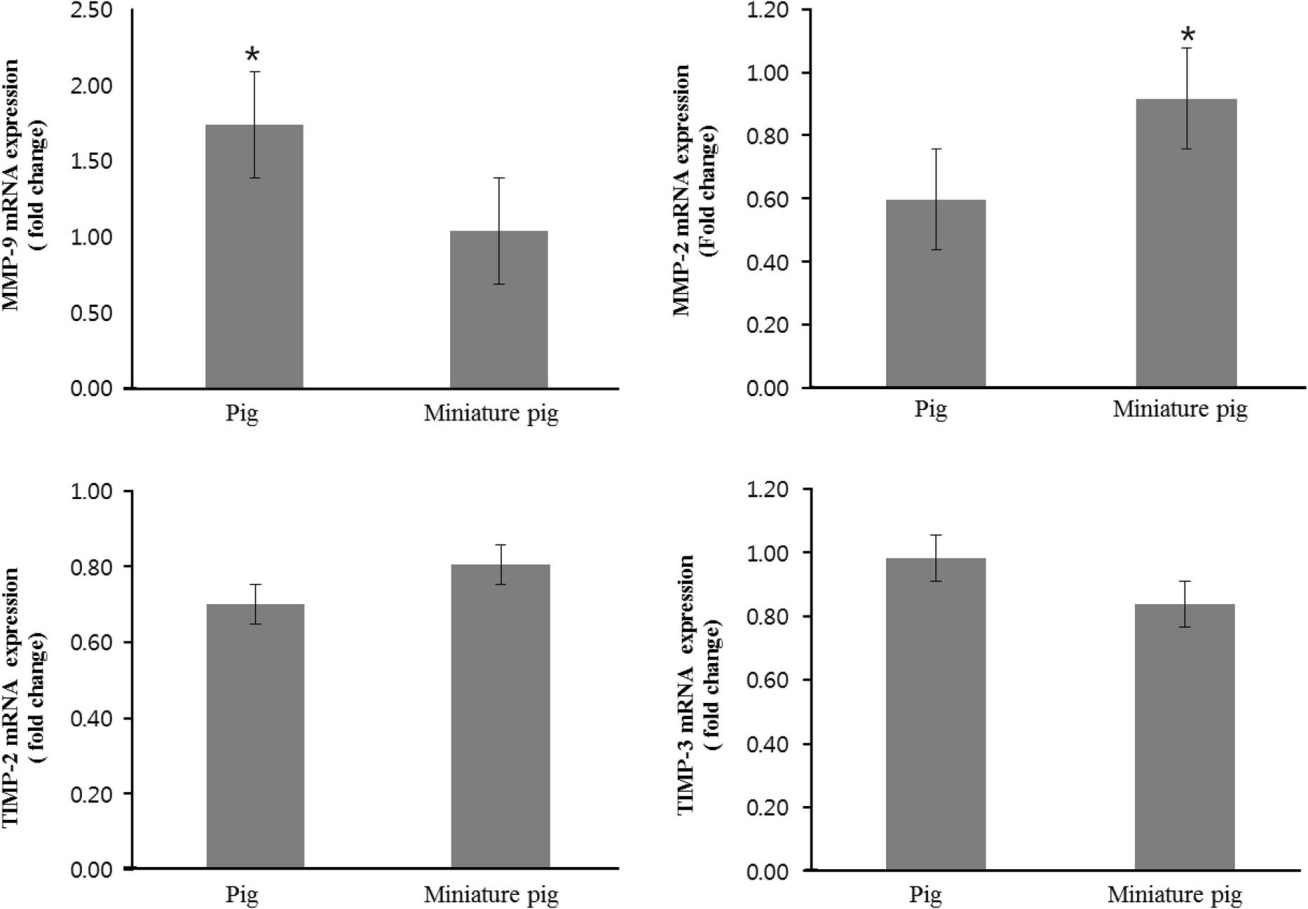
mRNA expression of MMP-2, MMP-9, TIMP-2, and TIMP-3 in the ovaries of normal and miniature pigs. mRNA levels were assessed by real time RT-PCR. The data are presented as average fold changes (mean ± SD) of three independent experiments. * *P* < 0.05

The mRNA expression of MMP-2, MMP-9, TIMP-2, and TIMP-3 in the ovaries of normal and miniature pigs is shown in Fig. 1. The expression of MMP-9, which promotes follicle development by regulating FSH, was higher in normal pigs than in miniature pigs. On the other hand, the level of TIMP-3 mRNA (MMP-9 inhibiting factor) in the ovaries was higher for miniature pigs than for normal pigs. The expression of MMP-2 mRNA was more in the ovaries of miniature pigs, whereas that of TIMP-2 mRNA was higher in normal pigs.

### Changes in gelatinase activity during follicle development

**Fig. 2:**
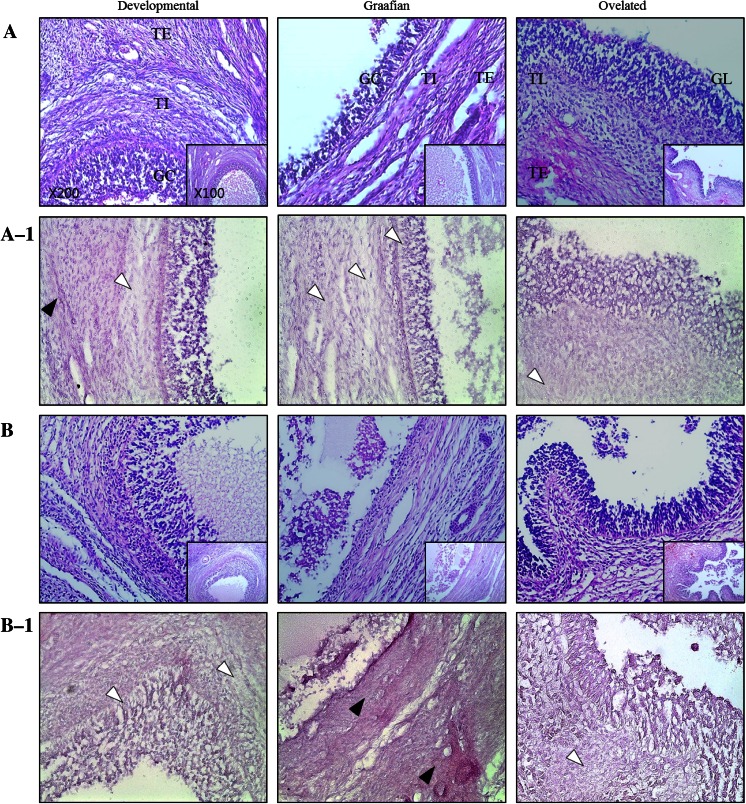
In-situ zymography analysis of gelatinase activity during follicular development in normal and miniature pigs. H&E staining of follicles in normal pigs (**A**) and miniature pigs (**B**); in situ zymography for normal pigs (**A-1**) and miniature pigs (**B-1**). *Black arrows* no active MMPs, *white arrows* active MMPs. *TE* Theca externa cells, *TI* theca interna cells, *GC* granulosa cell, *TL* theca lutein cells, *GL* granulosa lutein cells. Original magnification ×200

Gelatinase activity in the ovaries of normal and miniature pigs was evaluated using in situ zymography (Fig. 2). Overall, gelatinase activity was higher in the ovaries of normal pigs than in those of miniature pigs. During follicular development, similar gelatinase activity was observed in both breeds. In the Graafian follicles, gelatinase activity was high in normal pigs, especially in theca interna cells (Fig. 2A-1); in contrast, in miniature pigs, gelatinase activity was insignificant, with minimal activity in theca interna cells (Fig. 2B-1). Although gelatinase activity decreased after ovulation, it was still higher in normal pigs than in miniature pigs. Compared to high gelatinase activity in the Graafian follicles and theca interna, the activity observed in the theca externa was relatively low. After ovulation, low gelatinase activity was detected in the granulosa lutein cells of the luteinising follicles.

### MMP and TIMP expression in normal and miniature pigs

**Fig. 3:**
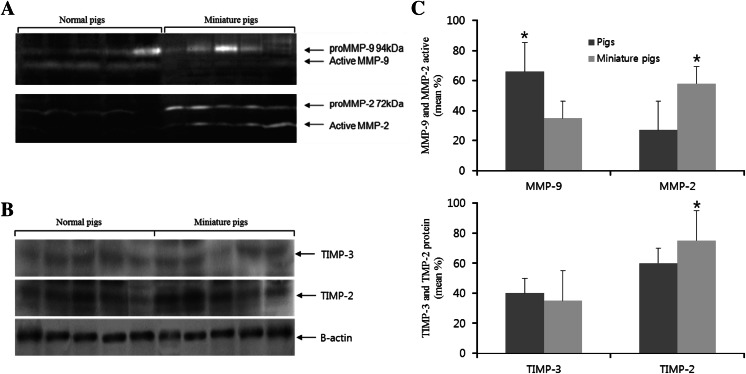
MMP activity and TIMP expression in the ovaries of normal and miniature pigs. Zymography analysis of the MMP activity in the ovaries (**a**). TIMP-2 and TIMP-3 protein expression by western blot (**b**). Bar graphs (*right*) show quantification of the detected bands by densitometry (**c**). Data are presented as the mean ± SEM of three independent experiments; values normalised to those of β-actin (a house-keeping gene)

The expression pattern of MMPs and TIMPs in the ovaries of normal and miniature pigs (five samples each) was analysed (Fig. 3). The expression of active MMP-9 was higher in the ovaries of normal pigs than in those of miniature pigs. On the other hand, the expression of pro-MMP-9 was higher in the ovaries of miniature pigs. The expression of pro-MMP-2 and active MMP-2 was lower in normal than in miniature pigs (Fig. 3a). For both pig breeds, the expression of TIMP-3 (MMP-9 inhibitor) was low and that of TIMP-2 (MMP-2 inhibitor) was high (Fig. 3b).

In situ expression and localisation of MMP-2, MMP-9, TIMP-2, and TIMP-3 in different stages of follicle development

**Fig. 4:**
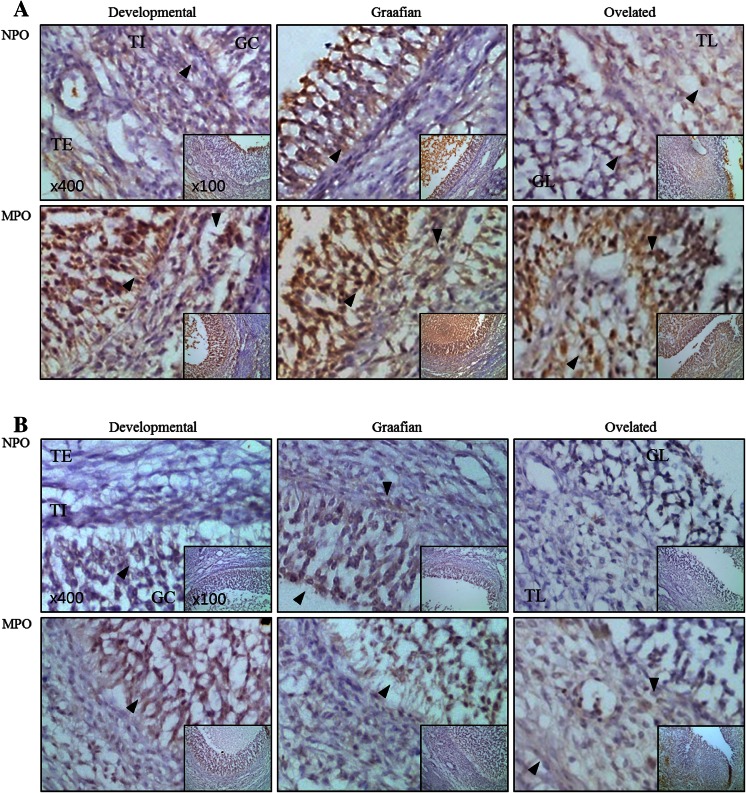

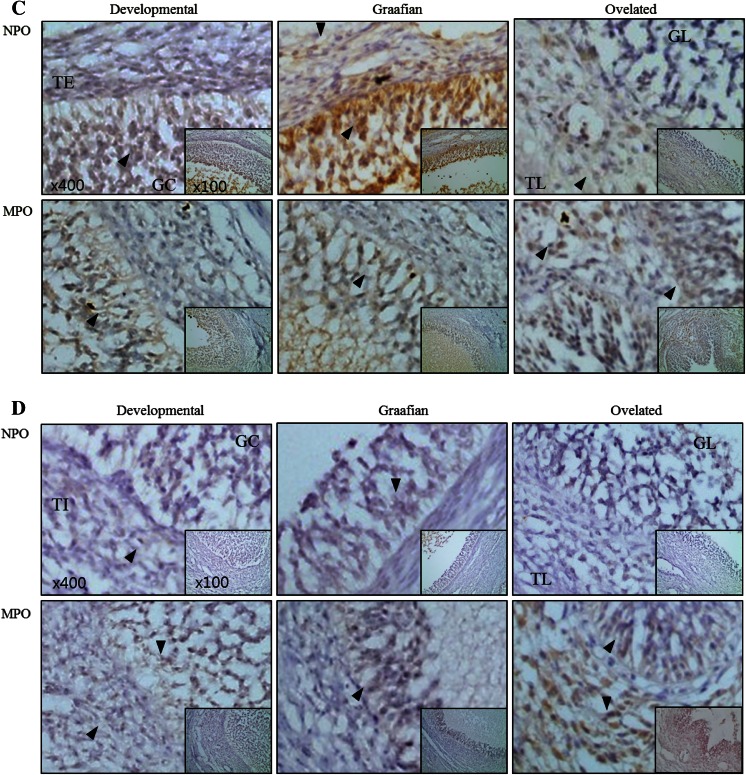
Expression and localisation of MMP-2 protein during follicular stage in normal and miniature pigs. Tissue sections of normal and miniature pig follicles were immunostained with the MMP-2 antibody and counterstained with H&E. *Black arrows* indicate MMP-2-expressing cells. **a** MMP-2, **b** TIMP-2, **c** MMP-9, **d** TIMP-3. *NPO* normal pig ovary, *MPO* miniature pig ovary, *TE* theca externa cells, *TI* theca interna cells, *GC* granulosa cells, *TL* theca lutein cells, *GL* granulosa lutein cells. Original magnification ×400 (inset ×100)

The protein expression patterns and localisation in each stage of follicle development are shown in Fig. 4. MMP-2 was mostly detected in the granulosa cells of normal pigs and was highly expressed in the theca externa of the developing follicles. During follicle development, higher levels of MMP-2 were observed in miniature pigs than in normal pigs. During this period, MMP-2 expression was low in theca externa cells, but high in theca interna and granulosa cells. In the Graafian follicles, MMP-2 expression was higher in miniature pigs than in normal pigs, both in theca interna and granulosa cells. MMP-2 was also highly expressed in theca lutein and granulosa lutein cells of the ovulated follicles (Fig. 4a).

A low level of the MMP-2 inhibitor TIMP-2 was observed in all ovarian cell types of normal and miniature pigs, except in the theca externa. In the theca interna of the developing follicles, TIMP-2 expression was higher in miniature pigs, whereas in the granulosa cells of the Graafian follicles, it was higher in normal pigs. After ovulation, TIMP-2 expression was relatively lower in the ovulated follicles of normal pigs than in those of miniature pigs. In miniature pigs, TIMP-2 was strongly expressed in theca lutein cells, while expression was lower in granulosa lutein cells (Fig. 4b).

Although MMP-9 was expressed in the granulosa cells of the developmental follicles of normal pigs, its expression was lower in the theca interna and externa. MMP-9 was highly expressed in all cells of the Graafian follicle, especially in the granulosa cells. Expression was low in the granulosa after ovulation but was high in the theca lutein and theca externa. MMP-9 was also highly expressed in the Graafian follicles of normal pigs. Overall, during follicular development MMP-9 expression was lower in all examined tissues of miniature pigs compared to those of normal pigs. In the granulosa lutein of ovulated follicles, the expression pattern was similar in both breeds, while in the theca lutein cells, a slightly higher expression was noted in miniature pigs (Fig. 4c).

The expression of the MMP-9 inhibitor TIMP-3 was not as strong as that of TIMP-2 in both breeds. Nevertheless, TIMP-3 expression in the theca interna of the developing follicles was higher in miniature pigs than in normal pigs, whereas in the Graafian follicles, it was similar for both groups. After ovulation, TIMP-3 expression was higher in all types of follicular cells (theca externa, theca lutein, and granulosa lutein) in miniature pigs. Overall, TIMP-3 was more prominently expressed in miniature pigs than in normal pigs (Fig. 4d).

## Discussion

Fluctuations in the levels of endocrine hormones affect the function of granulosa and theca cells, which play an important role in egg maturation (Hirshfield 1991). During maturation, follicles are re-structured by the changes in the ECM, and ovulation occurs at the Graafian stage of follicular development. MMPs are involved in follicular growth and maturation (Greenwald and Roy 1994) their expression patterns are regulated by oestrogen and progesterone (Riley et al. 2001). However, follicle development and egg maturation differ between normal and miniature pigs; miniature pigs have low ovulation rate and small litter size (Anderson 1997). In this study, we examined the influence of MMPs on follicular development by comparing the expression patterns of MMP-2 and MMP-9 in developing, Graafian, and ovulated follicles between normal and miniature pigs. We found that the expression of MMP-9, which induces follicle maturation, was more prominent in the ovaries of normal pigs, where Graafian follicles were abundant. On the other hand, expression of MMP-2, which affects reorganization of ovarian cells, was high in miniature pigs (Imai et al. 1999). We believe that the differential expression of MMP-9 and MMP-2 in the two swine breeds may provide an insight into the molecular basis of the reduced fertility in miniature pigs.

In both normal and miniature pigs, the activity of gelatinases tended to be low in developing follicles; however, it was strongly expressed in the Graafian follicles of normal pigs. These results are consistent with the findings of Curry and Osteen (2001). Gelatinase activity was also detectable in the Graafian follicles of miniature pigs; however, it was lower than that in normal pigs. After ovulation, gelatinase activity was higher in the ovulated follicles of normal pigs than in those of miniature pigs. Gelatinase activity was higher in ovulated follicles than in developing and Graafian follicles. Since gelatinase activity remained high during ovulation, it can be speculated that the surge in luteinising hormone (LH) levels triggers rapid ECM restructuring and apoptosis in follicular cells. During this process, ruptured follicles are effectively reorganised into the corpus luteum (Hagglund et al. 2001). It is likely that both relative paucity of follicular maturation-promoting MMP-9 and elevated expression of apoptosis-inducing MMP-2 may contribute to the compromised fertility of miniature pigs.

Curry and Osteen (2001) reported that MMP-2 and MMP-9 were expressed in the developing follicles, where major diffusion of granulosa cells occurs. Chaffin and Stouffer (1999) also reported that the gelatinase activity of MMP-2 and MMP-9 was required for the modification of granulosa cells during follicle development. In this regard, the reduction in the ovulation rate in miniature pigs may be due to the low expression of MMP-9 in granulosa cells. Higher MMP-2 expression in the Graafian follicles of miniature pigs could not compensate for the lack of MMP-9, probably because MMPs are required before the LH level surge to ensure successful ovulation (Espey and Lipner 1994).

Based on the results observed in our study, MMP-9 appears to be more important than MMP-2 for the structural changes in granulosa cells. Decreased expression of TIMP-2 (MMP-2 inhibitor) and increased expression of TIMP-3 (MMP-9 inhibitor) observed in miniature pigs lead to the inhibition of MMP-9 and enhancement of MMP-2 activity and have been shown to correlate with induction of ovulation and transformation of follicular cells into luteal cells (Ray and Stetler-Stevenson 1994), thus supporting our hypothesis. Other reports have also suggested that MMP-9 is necessary for the reorganization of follicles (Curry and Osteen 2001). On the other hand, several studies demonstrated the effect of MMP-2 on follicular development. It can be speculated that the reorganization of ovarian cells in normal and miniature pigs could be modulated by different MMPs. Taken together, our results suggest that the differential expression of the gelatinases MMP-2 and MMP-9, which degrade the basement membrane and promote reorganisation of ovarian cells, may contribute to the differences in the rate of ovulation, and follicle development between normal and miniature pigs.

## Conclusions

Our results reveal the difference in the expression patterns of MMPs and TIMPs during follicle development between normal and miniature pigs. MMP-9 was highly expressed in the Graafian follicles of normal pigs, whereas in miniature pigs, its levels during the follicular stage were lower than that in normal pigs. However, during the developing stages, MMP-2 expression was higher in miniature pigs than in normal pigs. TIMP-2 expression was low in both breeds, but TIMP-3 levels were higher in miniature pigs than in normal pigs. Together, these results indicate that MMP expression can be used as an indicator of developmental changes in porcine follicular cells. Based on MMP and TIMP expression profiles, we believe that MMPs play an important role in follicular development by regulating the remodelling of porcine follicular cells.

## Electronic supplementary material

Below is the link to the electronic supplementary material.

Supplementary material 1 (DOCX 13 kb)
