# Supplementary material for: Matrix metalloproteinases are important for follicular development in normal and miniature pigs
Source: Biotechnol Lett. 2014 Feb 22;36(6):1187–96. doi: 10.1007/s10529-014-1474-9 (PMC4000628; doi:10.1007/s10529-014-1474-9)
Supplement: Supplementary file 1 — Supplementary material 1 (DOCX 13 kb) [file 10529_2014_1474_MOESM1_ESM.docx]

**Supplementary Table 1.** Primers for real time RT-PCR analysis of the MMP and TIMP genes

| **Primer name** | **Sequence** | **Amplicon size** | **Gene ID** |
| --- | --- | --- | --- |
| GAPDH Fw | *5' CCCGTTCGACAGACAGCCGTG 3'* | 238bp | NM_001206359.1 |
| GAPDH Rv | *5' CCGCCTTGACTGTGCCGTGG 3'* |  |  |
| MMP-2 Fw | *5' TTCCCGGAGATGTCGCCCCC 3'* | 160bp | NM_214192.1 |
| MMP-2 Rv | *5' CCTGTCTGGGGGAGCCCGAA 3'* |  |  |
| MMP-9 Fw | *5' CAAACCACGCCAGCCCACCT 3'* | 147bp | NM_001038004.1 |
| MMP-9 Rv | *5' GCCGGTTCCAGGGACTGCTTT 3'* |  |  |
| TIMP-2 Fw | *5' GGTAGTGATCAGGGCCAA3 '* | 106bp | NM_001145985.1 |
| TIMP-2 Rv | *5' CTTTATCTGCTTGATCTCA 3'* |  |  |
| TIMP-3 Fw | *5' CTCCGACATCGTGATCCGGGC 3'* | 130bp | XM_003126073.3 |
| TIMP-3 Rv | *5' CTGCACATGGGGCATCTTGGTGA 3'* |  |  |

**MMP, matrix metalloproteinase; TIMP, tissue inhibitor of metalloproteinase**
